# Influence of Thermophoretic Particle Deposition on the 3D Flow of Sodium Alginate-Based Casson Nanofluid over a Stretching Sheet

**DOI:** 10.3390/mi12121474

**Published:** 2021-11-29

**Authors:** Bheemasandra M. Shankaralingappa, Javali K. Madhukesh, Ioannis E. Sarris, Bijjanal J. Gireesha, Ballajja C. Prasannakumara

**Affiliations:** 1 Department of Studies and Research in Mathematics, Kuvempu University, Shankaraghatta, Shimoga 577451, India; shankar.gsch@gmail.com (B.M.S.); bjgireesu@gmail.com (B.J.G.); 2 Department of Mathematics, Government Science College (Autonomous), Hassan 573201, India; 3 Department of Studies and Research in Mathematics, Davangere University, Davangere 577002, India; madhukeshjk@gmail.com (J.K.M.); dr.bcprasanna@gmail.com (B.C.P.); 4 Department of Mechanical Engineering, University of West Attica, 12244 Athens, Greece

**Keywords:** non-linear stretching sheet, Casson fluid: nanofluid, thermophoretic particle deposition

## Abstract

The wide range of industrial applications of flow across moving or static solid surfaces has aroused the curiosity of researchers. In order to generate a more exact estimate of flow and heat transfer properties, three-dimensional modelling must be addressed. This plays a vital role in metalworking operations, producing plastic and rubber films, and the continuous cooling of fibre. In view of the above scope, an incompressible, laminar three-dimensional flow of a Casson nanoliquid in the occurrence of thermophoretic particle deposition over a non-linearly extending sheet is examined. To convert the collection of partial differential equations into ordinary differential equations, the governing equations are framed with sufficient assumptions, and appropriate similarity transformations are employed. The reduced equations are solved by implementing Runge Kutta Fehlberg 4th 5th order technique with the aid of a shooting scheme. The numerical results are obtained for linear and non-linear cases, and graphs are drawn for various dimensionless constraints. The present study shows that improvement in the Casson parameter values will diminish the axial velocities, but improvement is seen in thermal distribution. The escalation in the thermophoretic parameter will decline the concentration profiles. The rate of mass transfer, surface drag force will reduce with the improved values of the power law index. The non-linear stretching case shows greater impact in all of the profiles compared to the linear stretching case.

## 1. Introduction

The vast range of technological applications of flow across moving or static solid surfaces has aroused the curiosity of researchers. The vaporisation of liquid coatings, the pulling of filaments through a static liquid, crystallization process techniques, the production of rubber and plastic films, and the nonstop cooling of fibre use these concepts. The research community has been studying various elements of such flows since the emergence of boundary layer models. Vajravelu [1] introduced the classical problem of two-dimensional motion caused by a non-linearly extending surface. Three-dimensional (3D) modelling must be considered to obtain a more precise estimation of flow and thermal transfer characteristics. As a result, many researchers have focused their efforts on studying three-dimensional flows. Recently, Gireesha and Umeshaih [2] examined the consequence of non-linear thermal radiation on a magnetohydrodynamic (MHD) 3D flow of Jeffery nanoliquid flow over a non-linearly porous extending sheet. The statistical analysis of a three-dimensional MHD convective Carreau nanofluid flow caused by a bidirectional non-linear stretching sheet with a heat source and zero mass flux was swotted by Sabu et al. [3]. Puneeth et al. [4] utilized the revised Buongiorno’s model features to inspect the 3D mixed convection flow of hybrid Casson nanoliquid via a non-linear extending surface. Khan et al. [5] investigated the solar energy applications in a 3D flow in a non-linear extending surface in the presence of nano liquid.

Non-Newtonian liquids are those that do not follow Newton’s viscosity rule. Shampoos, sauces, pastes, acrylics, colloidal solutions, and ketchup are just a few examples. Because of its rheological uses in chemical and mechanical engineering processes, most fluid mechanics researchers currently focus on this type of fluid. Casson liquid is one of the best non-Newtonian liquids because of its numerous applications in metallurgy, food dispensing, bioengineering, and penetrating activities. Casson liquid is classified as a shear depleting liquid with an indeterminable thickness at zero shear rate. Recently, Gowda et al. [6] examined the heat and mass transfer of the Marangoni driven boundary layer flow of a non-Newtonian nanofluid by considering the effects of binary chemical reactions and activation energy. Dahab et al. [7] investigated the MHD Casson nanofluid movement over a non-linearly warmed porous medium with suction/injection in the presence of an expanding surface effect. Madhukesh et al. [8] considered the Casson nanoliquid bio-Marangoni convection motion through a porous medium in the presence of chemically reactive activation energy. Kumar et al. [9] scrutinized the consequence of particle deposition on heat and mass transmission in Casson fluid motion over a moving thin needle. Jamshed et al. [10] deliberated the unsteady Casson nanoliquid flow on a stretching sheet in the presence of solar thermal radiation.

One of the most challenging difficulties that engineers and companies face is thermal management. The heat is transferred via base liquids. Heat distribution is a problem with these basic liquids. Researchers will add nanoparticles to these common base liquids to increase thermal dispersion and thermal distribution, and obtained fluids are called as nanoliquid. Nanoparticles often comprise oxides, metals, and carbon nanotubes with diameters ranging from 1 to 100 nm. Due to the augmentation in the rate of heat transfer, researchers will pay attention to nanofluids. Recently, Kumar et al. [11] examined the single-walled carbon nanotube (SWCNT)/multi-walled carbon nanotube (MWCNT) in a Maxwell nanofluid over a stretching surface in the presence of a magnetic dipole. Using a metaheuristic technique, Prasannakumara [12] conferred the investigation of nanofluid flow in a porous medium by considering local thermal non-equilibrium condition. Kumar et al. [13] studied the convective thermal distribution by utilizing the Koo–Kleinstreuer–Li (KKL) model for nanofluid flow over a coiled sheet. Karvelas et al. [14] pondered the computational analysis of paramagnetic spherical iron-oxide nanosized particles in the presence of permanent magnetic fields. Ramesh et al. [15] elaborated the importance of aluminium alloy particles suspension on fluid flow past parallel plates.

Thermophoresis is a phenomenon that happens when particles separated in vapor are subjected to a thermal gradient and migrate from the hot to the cold zone of the gas. Thermophoresis particles are used in a wide range of engineering and manufacturing purposes, including nuclear reactor safety, thermal precipitation design, and physical vapor confession. Chen et al. [16] studied the thermophoretic particle deposition (TPD) in a dual stratified Casson fluid in the presence of magnetic dipole. Alhadhrami et al. [17] swotted the influence of TPD in the presence of nano liquid flow over a wall jet by considering slip effects. Hafeez et al. [18] conferred the TPD and Soret-Dufour effects on a flow of liquid past a rotating disk. Ashraf et al. [19] scrutinized the numerical simulation of variable thermal conductivity and particle deposition effects on free convection over a sphere surface.

Based on the above served literature, the primary aim of the present article is to numerically analyze the thermal and mass distribution of a three-dimensional non-linear extending surface with aluminum oxide based Casson nanofluid in the presence of thermophoretic particle deposition, which has not yet been studied to the best of the authors’ knowledge. Thermophoretic particle deposition facilitates the investigation of differences in mass transfer performance due to modest aluminum oxide percentage changes. The present study is useful in various engineering applications. The governing equations that represent the flow and thermal characteristics with appropriate boundary conditions are formulated. These equations are reduced with suitable similarity variables and numerically solved by using an apt numerical method. Since many analytical and semi-analytical procedures exist to solve these kinds of equations, we used the RKF-45 method to fulfill this gap. The parameters that influence the flow characters are analyzed with respective profiles, and detailed discussions are made for both linear and non-linear cases. The essential engineering aspects are discussed.

## 2. Mathematical Model

Consider an incompressible, laminar 3D flow of a Casson nanoliquid in the presence of TPD over a non-linearly stretching sheet. The sheet is moving with uniform velocity $u_{w}=v_{w}={\left(x+y\right)}^{n}a$ in x&y directions, respectively, with a, n>0. $T_{w}$ and $C_{w}$ representing the wall temperature, and concentration as well as $T_{\infty }$ and $C_{\infty }$ denoting ambient temperature and concentration. Both $T_{w}$ and $C_{w}$ are assumed to be constant on the stretching surface. The ambient values temperature and concentration are denoted by $T_{\infty }$ and $C_{\infty }$ as the value of z→∞. Further, temperature and concentration at the wall are more significant than the ambient temperature and concentration.

The rheological equation of state for an isotropic and incompressible flow of Casson fluid is given by:

$$\tau_{ij}=\left\{\begin{array}{c} 2\left(\mu_{B}+p_{y}/\sqrt{2\pi }\right)e_{ij},\pi >\pi_{c},\\ 2\left(\mu_{B}+p_{y}/\sqrt{2\pi_{c}}\right)e_{ij},\pi <\pi_{c}. \end{array}\right.$$

In the above equation, π is the product of the deformation rate component and itself; i.e., $\pi =e_{ij}e_{ij}$ and $e_{ij}$ is the ${\left(i,j\right)}^{th}$ component of the deformation rate. $\pi_{c}$ is the critical value of this product based on the non-Newtonian model. $\mu_{B}$ is the plastic dynamic viscosity of the non-Newtonian fluid, and $p_{y}$ signifies the yield stress of the fluid.

**Figure 1:**
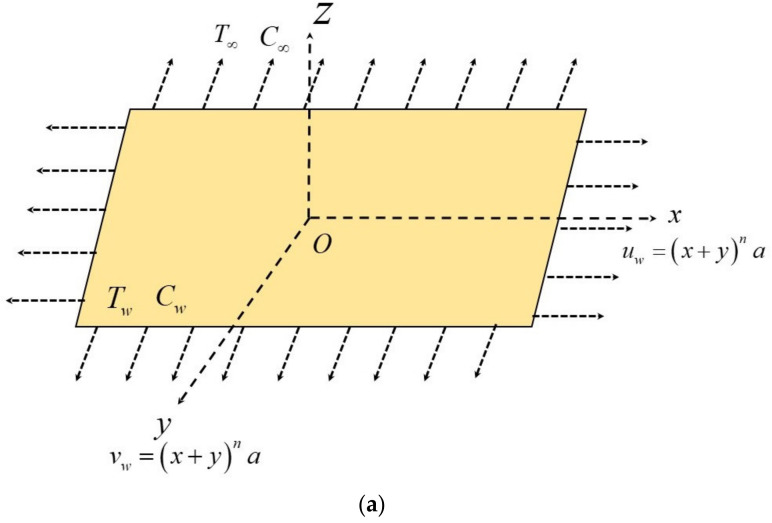

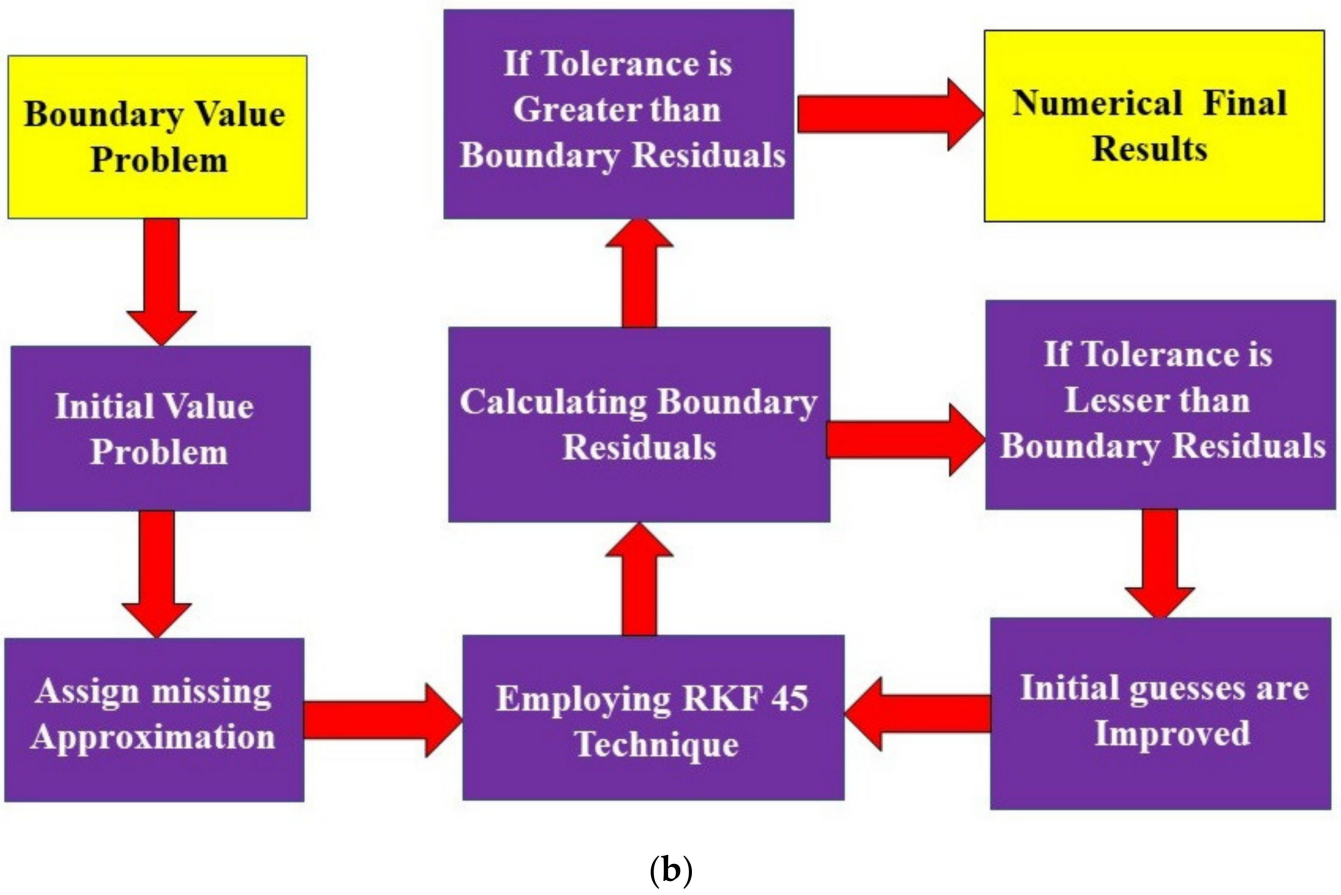
(**a**): Physical representation and coordinate system. (**b**) Flow chart for numerical scheme.

The geometry of the described problem is illustrated in Figure 1a. Based on the above assumptions, the governing equations and boundary conditions are given (see Epstein et al. [20], Butt et al. [21], Raju et al. [22], Khan et al. [23]).

(1) $$u_{x}+v_{y}+w_{z}=0$$

(2) $$uu_{x}+vu_{y}+wu_{z}=\left(1+\frac{1}{\beta }\right)\nu_{nf}u_{zz}$$

(3) $$uv_{x}+vv_{y}+wv_{z}=\left(1+\frac{1}{\beta }\right)\nu_{nf}v_{zz}$$

(4) $$uT_{x}+vT_{y}+wT_{z}=\frac{k_{nf}}{{\left(\rho Cp\right)}_{nf}}T_{zz}$$

(5) $$uC_{x}+vC_{y}+wC_{z}=D_{nf}C_{zz}-{\left(V_{T}\left(C-C_{\infty }\right)\right)}_{z}$$

Boundary conditions (see Raju et al. [22], Khan et al. [23])

(6) $$u=u_{w},T=T_{w},v=v_{w},C=C_{w},w=0\;\mathrm{at}\;z=0$$

(7) $$C\to C_{\infty },u\to 0,v\to 0,T\to T_{\infty }\;\mathrm{as}\;z\to \infty$$

From the Equations (1)–(7), $u,v,w\left({\mathrm{ms}}^{-1}\right)$ are the velocity components along the $x,y,z\left(m\right)$ directions, respectively. $\beta \left(-\right)$ is the Casson parameter, $\nu =\left(\frac{\mu }{\rho }\right)\left(m^{2}s^{-1}\right)$ signifies the kinematic viscosity, $\mu \left({\mathrm{kgm}}^{-1}s^{-1}\right)$ signifies dynamic viscosity, $\rho \left({\mathrm{kgm}}^{-3}\right)$ is the density, $k\left({\mathrm{kgms}}^{-3}K^{-1}\right)$ signifies thermal conductivity, $Cp\left(m^{2}s^{-2}K^{-1}\right)$ signifies specific heat, $D\left(m^{2}s^{-1}\right)$ is diffusivity, and $V_{T}\left({\mathrm{ms}}^{-1}\right)$ is the thermophoretic velocity.

The term $V_{T}$ is defined as

(8) $$V_{T}=-\left(\frac{K_{1}\nu_{nf}}{T_{r}}\right)T_{z}$$

Here, $K_{1}$ is the thermophoretic coefficient and $T_{r}$ is the reference temperature (see Epstein et al. [20]).

The below-mentioned similarity variables are introduced (see Raju et al. [22], Khan et al. [23]).

$$v=a{\left(x+y\right)}^{n}g\prime \left(\eta \right),w=-\left(\left(f\prime \left(\eta \right)+g\prime \left(\eta \right)\right)\left(\frac{n-1}{2}\right)\eta +\left(f\left(\eta \right)+g\left(\eta \right)\right)\left(\frac{n+1}{2}\right)\right){\left(x+y\right)}^{\frac{n-1}{2}}\sqrt{a\nu_{f}},$$

(9) $$u=a{\left(x+y\right)}^{n}f\prime \left(\eta \right),\eta ={\left(x+y\right)}^{\frac{n-1}{2}}\sqrt{\frac{a}{\nu_{f}}}z,\;T-T_{\infty }=\theta \left(\eta \right)\left(T_{w}-T_{\infty }\right),C=C_{\infty }+\chi \left(\eta \right)\left(C_{w}-C_{\infty }\right).$$

After substituting Equations (8) and (9) into (1)–(6) and simplifying, the following equations are obtained.

(10) $$\left(1+\frac{1}{\beta }\right)f\prime \prime \prime +\varsigma_{1}\varsigma_{2}\left(\left(f+g\right)\left(\frac{n+1}{2}\right)f\prime \prime -nf\prime \left(f\prime +g\prime \right)\right)=0$$

(11) $$\left(1+\frac{1}{\beta }\right)g\prime \prime \prime +\varsigma_{1}\varsigma_{2}\left(\left(f+g\right)\left(\frac{n+1}{2}\right)g\prime \prime -ng\prime \left(f\prime +g\prime \right)\right)=0$$

(12) $$\frac{k_{nf}}{k_{f}}\theta \prime \prime +Pr\varsigma_{3}\left(\theta \prime \left(\frac{n+1}{2}\right)\left(f+g\right)\right)=0$$

(13) $$\varsigma_{1}\chi \prime \prime +Sc\chi \prime \left(\frac{n+1}{2}\right)\left(f+g\right)-\left(\theta \prime \prime \chi +\chi \prime \theta \prime \right)\tau Sc=0$$

with

(14) $$\left.\begin{array}{c} \left(f\prime ,g\prime ,f,g,\theta ,\chi \right)\left(0\right)=\left(1,1,0,0,1,1\right)at\eta =0\\ \left(f\prime ,g\prime ,\theta ,\chi \right)\left(\infty \right)=\left(0,0,0,0\right)as\eta \to \infty \end{array}\right\}$$

The proposed problem deals with two different cases based on the following considerations:

(1) n=1: Linear stretching.
(2) n>1: non-Linear stretching.

Where, $\varsigma_{1}={\left(1-\phi \right)}^{2.5}$, $\varsigma_{2}=\left(1-\phi +\phi \frac{\rho_{s}}{\rho_{f}}\right)$, $\varsigma_{3}=\left(1-\phi +\phi \frac{{\left(\rho Cp\right)}_{s}}{{\left(\rho Cp\right)}_{f}}\right)$, $Sc=\frac{\nu_{f}}{D_{f}}$ is the Schmidt number, $Pr=\frac{\mu_{f}Cp_{f}}{k_{f}}$ is the Prandtl number, and $\tau =-\frac{K_{1}\left(T_{w}-T_{\infty }\right)}{T_{r}}$ signifies thermophoretic parameter.

The thermophysical properties of nanofluid are given (see Khan et al. [24], Devi and Devi [25]):

(15) $$\left.\begin{array}{c} k_{nf}=\frac{-2\phi \left(-k_{s}+k_{f}\right)+2k_{f}+k_{s}}{2\phi \left(-k_{s}+k_{f}\right)+2k_{f}+k_{s}}k_{f},{\left(\rho Cp\right)}_{nf}=\left(1-\phi +\frac{{\left(\rho Cp\right)}_{s}}{{\left(\rho Cp\right)}_{f}}\phi \right){\left(\rho Cp\right)}_{f}\\ \mu_{nf}=\frac{\mu_{f}}{{\left(1-\phi \right)}^{2.5}},\rho_{nf}=\rho_{f}\left(1-\phi +\phi \frac{\rho_{s}}{\rho_{f}}\right),D_{nf}=D_{f}{\left(1-\phi \right)}^{2.5} \end{array}\right\}$$

### Engineering Coefficients

The expressions and reduced forms of skin friction along the x and y directions is given as follows (see Raju et al. [22], Khan et al. [23]):

(16) $$\mathrm{Along}x\mathrm{direction}:\;C_{fx}=\frac{\mu_{nf}\left(1+\frac{1}{\beta }\right){\left(u_{z}+w_{x}\right)}_{z=0}}{\rho_{f}u_{w}^{2}}\to \sqrt[{fx\left(1+\frac{1}{\beta }\right)\frac{f\prime \prime \left(0\right)}{\varsigma_{1}}}]{Re}$$

(17) $$\mathrm{Along}y\mathrm{direction}:\;C_{fy}=\frac{\mu_{nf}\left(1+\frac{1}{\beta }\right){\left(v_{z}+w_{y}\right)}_{z=0}}{\rho_{f}u_{w}^{2}}\to \sqrt[{fx\left(1+\frac{1}{\beta }\right)\frac{g\prime \prime \left(0\right)}{\varsigma_{1}}}]{Re}$$

(18) $$\mathrm{Nusselt}\;\mathrm{number}:\;Nu=\frac{-k_{nf}\left(x+y\right){\left(T_{z}\right)}_{z=0}}{k_{f}\left(T_{w}-T_{\infty }\right)}\to \frac{Nu}{\sqrt{Re}\frac{-k_{nf}\theta \prime \left(0\right)}{k_{f}}}$$

(19) $$\mathrm{Sherwood}\;\mathrm{number}:\;Sh=\frac{-D_{nf}\left(x+y\right){\left(C_{z}\right)}_{z=0}}{D_{f}\left(C_{w}-C_{\infty }\right)}\to \frac{Sh}{{\sqrt{Re}}_{1}\left(0\right)}$$

where, $Re=\frac{u_{w}\nu_{f}^{-1}}{{\left(x+y\right)}^{-1}}$ is the local Reynolds number.

## 3. Numerical Procedure

The set of reduced equations stated in Equations (10)–(13) and boundary constraints (14) are solved using the RKF-45 method and the shooting scheme. The obtained equations are higher-order and two-point in nature. To get the solution, we reduced the system of ODEs and boundary conditions into a first-order system by substituting $a_{1}=f,a_{2}=f\prime ,a_{3}=f\prime \prime ,\;a_{4}=g,a_{5}=g\prime ,$ $a_{6}=g\prime \prime ,a_{7}=\theta ,$ $a_{8}=\theta \prime ,a_{9}=\chi ,$ $a_{10}=\chi \prime$ and obtain

(20) $$\left[\begin{array}{c} a_{1}^{\prime }\\ a_{2}^{\prime }\\ a_{3}^{\prime }\\ a_{4}^{\prime }\\ a_{5}^{\prime }\\ a_{6}^{\prime }\\ a_{7}^{\prime }\\ a_{8}^{\prime }\\ a_{9}^{\prime }\\ a_{10}^{\prime } \end{array}\right]=\left[\begin{array}{c} a_{2}\\ a_{3}\\ \frac{-1}{\left(1+\frac{1}{\beta }\right)}\varsigma_{1}\varsigma_{2}\left(\left(\frac{n+1}{2}\right)\left(f+g\right)f\prime \prime -nf\prime \left(f\prime +g\prime \right)\right)\\ a_{5}\\ a_{6}\\ \frac{-1}{\left(1+\frac{1}{\beta }\right)}\varsigma_{1}\varsigma_{2}\left(\left(\frac{n+1}{2}\right)\left(f+g\right)g\prime \prime -ng\prime \left(f\prime +g\prime \right)\right)\\ a_{8}\\ -\frac{k_{f}}{k_{nf}}Pr\varsigma_{3}\left(\left(\frac{n+1}{2}\right)\left(f+g\right)\theta \prime \right)\\ a_{10}\\ \frac{1}{\varsigma_{1}}\left(\left(a_{8}^{\prime }\chi +\chi \prime \theta \prime \right)\tau Sc-Sc\left(\frac{n+1}{2}\right)\chi \prime \left(f+g\right)\right) \end{array}\right]$$

with,

(21) $$\left[\begin{array}{c} a_{1}\left(0\right)\\ a_{2}\left(0\right)\\ a_{3}\left(0\right)\\ a_{4}\left(0\right)\\ a_{5}\left(0\right)\\ a_{6}\left(0\right)\\ a_{7}\left(\infty \right)\\ a_{8}\left(\infty \right)\\ a_{9}\left(\infty \right)\\ a_{10}\left(\infty \right) \end{array}\right]=\left[\begin{array}{c} 0\\ 1\\ \lambda_{1}\\ 0\\ 1\\ \lambda_{2}\\ 1\\ \lambda_{3}\\ 1\\ \lambda_{4} \end{array}\right]$$

The IVP stated in Equations (20) and (21) are solved by the RKF-45 method, and unknown values are obtained by the shooting scheme by setting step size h=0.01 and error tolerance $10^{-6}$, which satisfy the boundary condition at infinity. The numerical solutions are obtained with build-in package bvp4c by MATLAB and setting the constraints’ values as ϕ=0.01,β=0.5,Pr=6.45,Sc=0.8&τ=0.1. Figure 1b shows the flow chart for the numerical scheme. The solutions are compared with the existing works, and they best match each other (see Table 1 and Table 2).

## 4. Results and Discussion

The fundamental goal of the present section is to analyze the influence of various dimensionless constraints on their respective profiles. The reduced ODEs (10–13) and boundary conditions (14) are numerically solved with RK-45 and shooting schemes. We convert the ODEs into IVP, and the shooting scheme is adopted to obtain the missing boundary conditions. Thermophysical properties of nanoparticle and base liquid are presented in Table 3. The numerical procedure is validated with the previously existing works. The obtained results show the influence of the dimensionless parameters, i.e., Casson parameter $\left(\beta \right)$, power-law index $\left(n\right)$, Schmidt number $\left(Sc\right)$, and thermophoretic parameter $\left(\tau \right)$, on the axial velocity profiles and the thermal and concentration profiles. Throughout the analysis, computations are made for the power-law index n=1&n=3.

**Figure 2:**
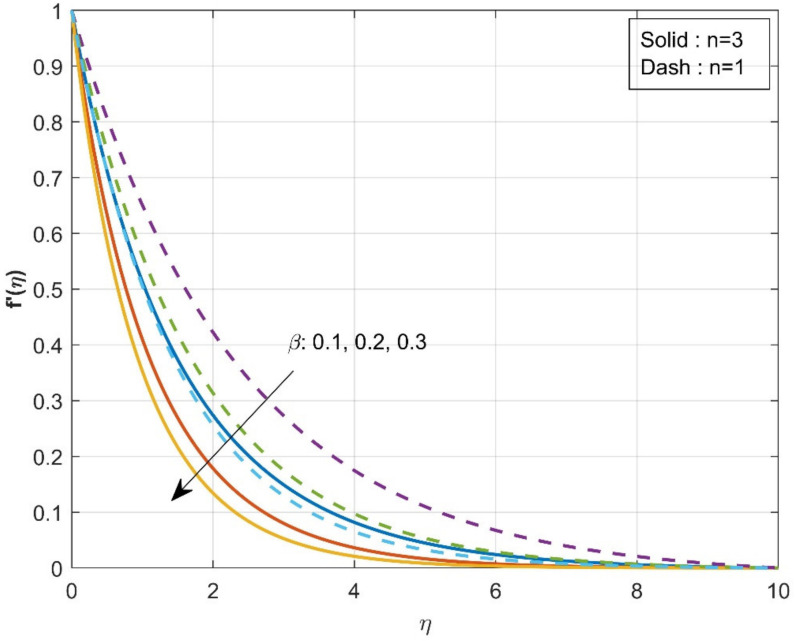
Influence of β over axial velocity $f\prime \left(\eta \right)$.

**Figure 3:**
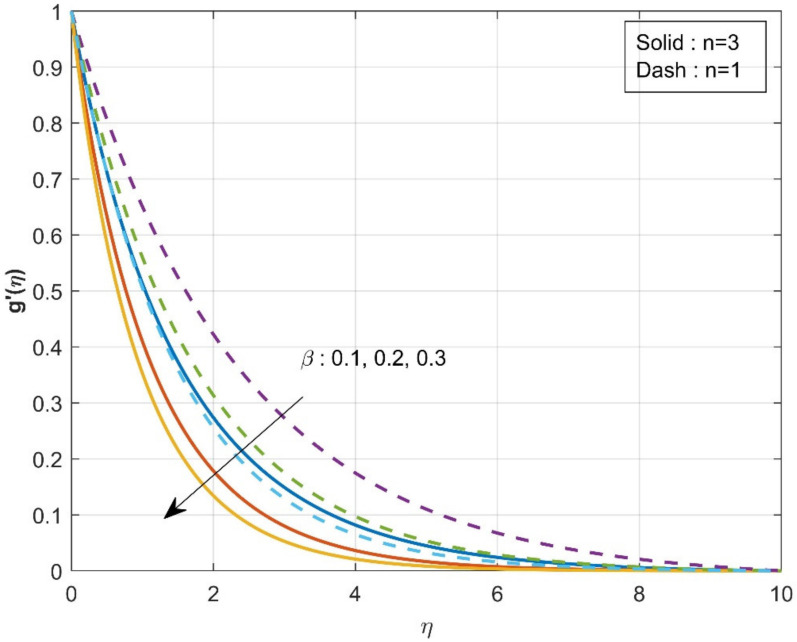
Influence of β over axial velocity $g\prime \left(\eta \right)$.

**Figure 4:**
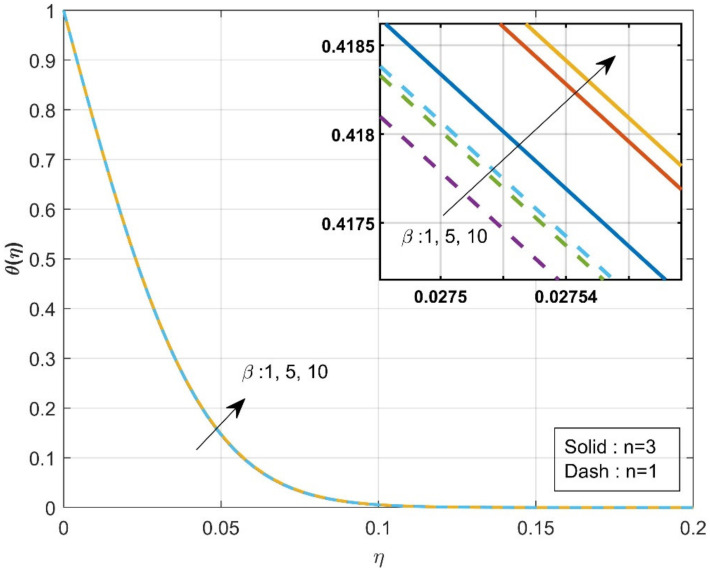
Influence of β over thermal profile $\theta \left(\eta \right)$.

**Figure 5:**
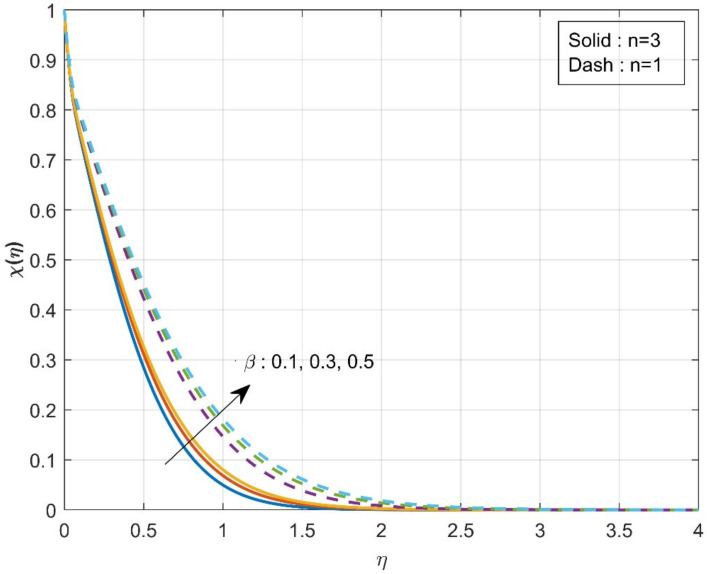
Influence of β over concentration $\chi \left(\eta \right)$.

Figure 2 estimates the influence of the β on axial velocity $f\prime \left(\eta \right)$. The rise in the values of the β will diminish $f\prime \left(\eta \right)$. From the physical point of view, increased $\beta \left(=0.1,0.2,0.3\right)$ values reduce fluid flow because the flow is subjected to a higher viscous force. It is further observed that axial velocity is diminished more in the case of n=3 than in the case of n=1. The influence of the β on axial velocity $g\prime \left(\eta \right)$ is displayed in Figure 3. A similar trend is observed as seen in the axial velocity profile $f\prime \left(\eta \right)$. The variation of the thermal distribution profile $\theta \left(\eta \right)$ for the rise in the values of the Casson parameter $\left(\beta \right)$ is portrayed in Figure 4. The rise in the values of β will enrich the thermal distribution in the system. In physical view, increasing the Casson parameter β increases the fluid’s boundary layer thickness, resulting in increased thermal distribution. The thermal distribution is more in the case of n=3 than in the case of n=1. Figure 5 is drawn to show the influence of the β on the mass transfer profile $\chi \left(\eta \right)$. The rise in values of Casson parameter will enhance the accumulation of particles, resulting in the enrichment of boundary layer thickness. As a result, the mass transfer enhances. The enhancement in concentration is more in the case of n=1 than in the case of n=3.

**Figure 6:**
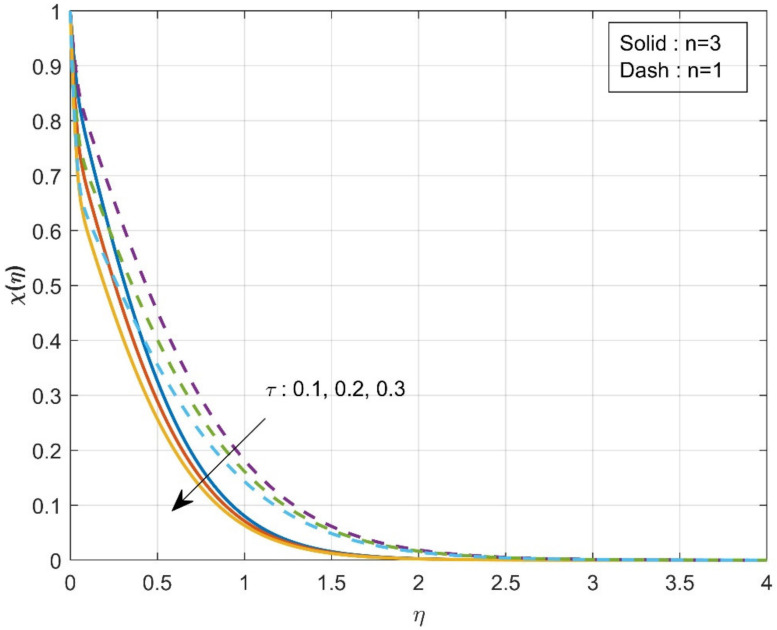
Influence of τ over concentration $\chi \left(\eta \right)$.

**Figure 7:**
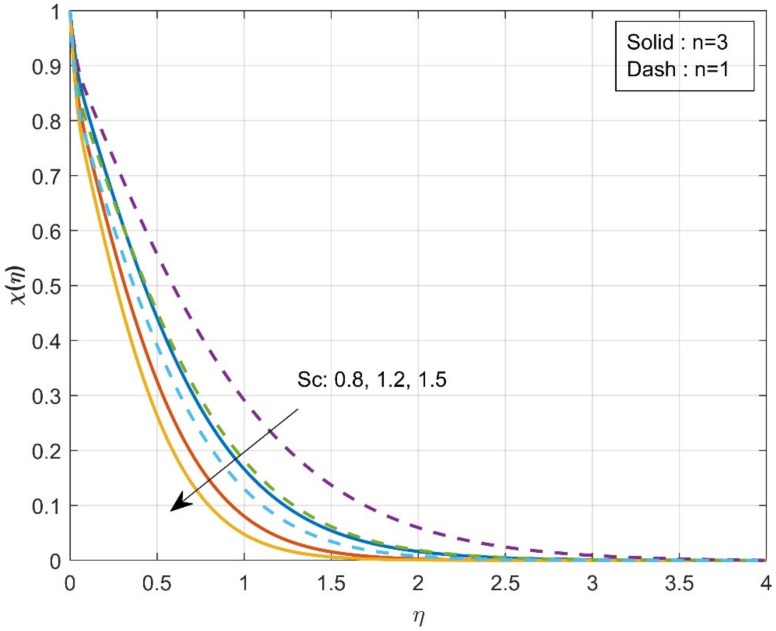
Influence of Sc over concentration $\chi \left(\eta \right)$.

**Figure 8:**
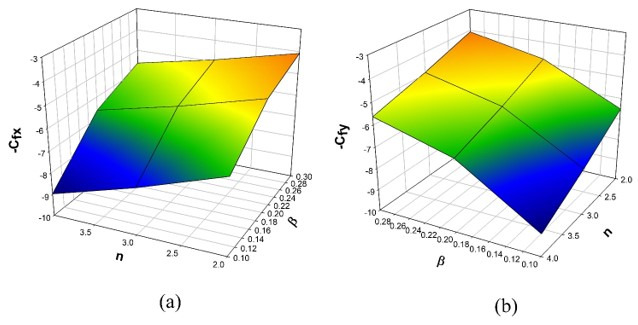
(**a**) Consequence of n and β over $C_{fx}$, (**b**) Consequence of n and β over $C_{fy}$.

**Figure 9:**
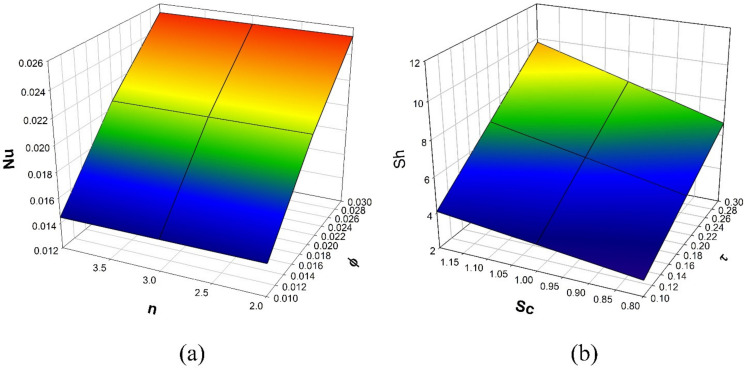
(**a**) Consequence of n and ϕ over Nu, (**b**) Consequence of Sc and τ over Sh.

The variation of τ on $\chi \left(\eta \right)$ is revealed in Figure 6. The enhancement in $\left(\tau \right)$ values will reduce concentration $\chi \left(\eta \right)$. The mobility of the particles increases as the temperature gradient grows, resulting in a decrease in fluid concentration. The influence of the Sc on the $\chi \left(\eta \right)$ is drawn in Figure 7. The concentration profile decreases as the Schmidt number increases. The Schmidt number is the physical definition of the kinematic viscosity to molecular diffusion coefficient ratio. As a result, the enhanced values of Sc decline $\chi \left(\eta \right)$. The significant concentration diminishes more in the case of n=3 than in the case of n=1 for τ and Sc. Figure 8a represents the variation of surface drag force in x direction on n for the various values of β. The rise in the values of β will weaken the surface drag force. This is due to the improvement in the values of β, and the n values will improve the thermal boundary layer and the liquid distribution. As a result, the surface drag will reduce. Similar behavior is seen in surface drag force along y direction (see Figure 8b). The variation of rate of thermal distribution on n for the numerous values of ϕ is shown in Figure 9a. The rise in the solid volume fraction will improve the boundary layer thickness and improve the thermal distribution rate. Figure 9b illustrates the consequence of the rate of mass transfer on Sc for the numerous values of τ. The rate of mass transfer will enhance with the increase in the thermophoretic parameter.

Table 4 is drawn to show the computational values of $f\prime \prime \left(0\right)$, $g\prime \prime \left(0\right)$, $\theta \prime \left(0\right)$, and $\chi \prime \left(0\right)$ over various dimensionless constraints. From the table, it is clear that increased values of n will diminish the surface drag coefficients $f\prime \prime \left(0\right)\&g\prime \prime \left(0\right)$ along x&y directions and mass transfer rate but will improve the rate of thermal distribution. The addition of solid volume fraction will diminish the $f\prime \prime \left(0\right)$, $g\prime \prime \left(0\right)$, $\theta \prime \left(0\right)$, and $\chi \prime \left(0\right)$ profiles. The increase in the values of β will reduce $f\prime \prime \left(0\right)\&g\prime \prime \left(0\right)$, but a reverse trend is seen for the $\theta \prime \left(0\right)\&\chi \prime \left(0\right)$ profiles. Improvement in the values of Sc&τ will decrease the $\theta \prime \left(0\right)\&\chi \prime \left(0\right)$ profiles.

## 5. Conclusions

An incompressible, laminar three-dimensional flow of a Casson nanoliquid in the presence of thermophoretic particle deposition over a non-linearly stretching sheet is examined. To convert the collection of partial differential equations into ordinary differential equations, the governing equations are constructed with appropriate assumptions, and acceptable similarity variables are employed. The simplified equations are solved using software by applying the Runge Kutta Fehlberg 4th 5th order method with a shooting scheme. The graphs are drawn for various constraints, and important engineering factors are discussed in detail. The significant findings of the current study are as follows:

1. Improvement in the Casson parameter will decline the axial velocity in x&y directions due to higher viscous force.
2. The thermal distribution is improved with enhancement in the Casson parameter due to an increment in boundary layer thickness.
3. Improved values of the Schmidt number will decline the concentration due to an increase in mass diffusivity.
4. An increase in the values of the thermophoretic parameter affects the concentration profiles due to an increment in the temperature gradient.
5. The rate of mass transfer will decrease with an upsurge in the values of the thermophoretic parameter.
6. The rate of thermal distribution will improve with an increment in the Casson parameter due to an enhancement in the thickness of the boundary layer.
7. The axial velocity and thermal distribution will be more in the case of n=3, but a reverse trend is perceived in the case of concentration profile.

## Figures and Tables

**Table 1:** Nanoparticle and base liquid thermophysical characteristics (see Khan et al. [24]).

Property	$C_{6}H_{9}NaO_{7}\left(SA\right)$	$Al_{2}O_{3}$
Pr	6.45	-
ρ	989	3970
Cp	4175	765
k	0.613	40

**Table 2:** Comparison of the model for $f\prime \prime \left(0\right)$ in the absence of $\phi \&\left(1+\frac{1}{\beta }\right)=1$.

n	Khan et al. [23]	Raju et al. [22]	Present Result
1	−1.414214	−1.4142141	−1.415192
3	−2.297186	−2.2971860	−2.297297

**Table 3:** Comparison of the model for $g\prime \prime \left(0\right)$ in the absence of $\phi \&\left(1+\frac{1}{\beta }\right)=1$.

n	Khan et al. [23]	Raju et al. [22]	Present Result
1	−1.414214	−1.4142140	−1.415192
3	−2.297186	−2.2971860	−2.297297

**Table 4:** Computational values of $f\prime \prime \left(0\right)$, $g\prime \prime \left(0\right)$, $\theta^{\prime }\left(0\right),$ and $\chi \prime \left(0\right)$ for various dimensionless constraints. When n=3,ϕ=0.01,β=0.1,and Sc=0.8&τ=0.1.

n	ϕ	β	Sc	τ	$-f\prime \prime \left(0\right)$	$-g\prime \prime \left(0\right)$	$-\theta \prime \left(0\right)$	$-\chi \prime \left(0\right)$
1					0.428707	0.428707	23.723047	2.883644
2					0.576782	0.576782	23.720796	3.072287
3	0.01	0.1	0.8	0.1	0.694439	0.694439	23.718549	3.231165
	0.01				0.694439	0.694439	23.718549	3.231092
	0.02				0.695638	0.695638	34.085641	4.164654
	0.03				0.696469	0.696469	42.056350	4.941831
		0.1			0.694439	0.694439	23.718549	3.231092
		0.2			0.940005	0.940005	23.712475	3.182007
		0.3			1.106068	1.106068	23.707350	3.148383
			0.8		0.694439	0.694439	23.718549	3.231092
			1.0		0.694439	0.694439	23.718549	3.873363
			1.2		0.694439	0.694439	23.718549	4.496217
				0.1	0.694439	0.694439	23.718549	3.231092
				0.2	0.694439	0.694439	23.718549	5.174942
				0.3	0.694439	0.694439	23.718549	7.125058

## Data Availability

Not applicable.

## Nomenclature

${\mathrm{Al}}_{2}O_{3}$	Aluminium oxide.
$C_{6}H_{9}{\mathrm{NaO}}_{7}\left(\mathrm{SA}\right)$	Sodium Alginate.
a	Stretching rate.
C	Concentration.
$C_{w}$	Concentration at wall.
$C_{\infty }$	Ambient concentration.
Cp	Specific heat.
$C_{fx}$	Skin friction.
D	Diffusivity.
k	Thermal conductivity.
$K_{1}$	Thermophoretic constant.
$f\left(\eta \right)\&g\left(\eta \right)$	Dimensionless velocity components.
n	Power law index.
Nu	Nusselt number.
Pr	Prandtl number.
Re	Local Reynolds number.
Sh	Sherwood number.
Sc	Schmidt number.
T	Temperature.
$T_{r}$	Reference temperature.
$T_{w}$	Wall temperature.
$T_{\infty }$	Ambient temperature.
$V_{T}$	Thermophoretic velocity.
Greek Symbols	
β	Casson parameter.
μ	Dynamic viscosity.
ρ	Density.
ν	Kinematic viscosity.
η	Similarity variable.
$\theta \left(\eta \right)\&\chi \left(\eta \right)$	Dimensionless temperature and concentration.
ϕ	Solid volume fraction.
τ	Thermophoretic parameter.
Subscripts:	
f	Fluid.
nf	Nanofluid.
S	Solid particle.
Abbreviations	
PDE	Partial differential equation.
ODE	Ordinary differential equation.
RKF-45	Runge Kutta Felhberg 4th 5th order.
TPD	Thermophoretic particle deposition.
MHD	Magneto hydrodynamic.
3D	Three dimensional.

## References

[1] Vajravelu K. (2001). Viscous flow over a non-linearly stretching sheet. Appl. Math. Comput..

[2] Jayanna G.B., Umeshaiah M., Prasannakumara B.C., Shashikumar S.N., Archana M. (2020). Impact of non-linear thermal radiation on magnetohydrodynamic three dimensional boundary layer flow of Jeffrey nanofluid over a non-linearly permeable stretching sheet. Phys. A Stat. Mech. Its Appl..

[3] Sabu A.S., Areekara S. (2021). Mathew, Statistical analysis on three-dimensional MHD convective Carreau nanofluid flow due to bilateral non-linear stretching sheet with heat source and zero mass flux condition. Heat Transfer..

[4] Puneeth V., Manjunatha S., Madhukesh J., Ramesh G. (2021). Three dimensional mixed convection flow of hybrid casson nanofluid past a non-linear stretching surface: A modified Buongiorno’s model aspects. Chaos Solitons Fractals.

[5] Khan J.A., Mustafa M., Hayat T., Alsaedi A. (2015). Three-dimensional flow of nanofluid over a non-linearly stretching sheet: An application to solar energy. Int. J. Heat Mass Transf..

[6] Gowda R.P., Kumar R.N., Jyothi A., Prasannakumara B., Sarris I. (2021). Impact of Binary Chemical Reaction and Activation Energy on Heat and Mass Transfer of Marangoni Driven Boundary Layer Flow of a Non-Newtonian Nanofluid. Processes.

[7] Abo-Dahab S.M., Abdelhafez M.A., Mebarek-Oudina F., Bilal S.M. (2021). MHD Casson nanofluid flow over non-linearly heated porous medium in presence of extending surface effect with suction/injection. Indian J. Phys..

[8] Madhukesh J.K., Ramesh G.K., Prasannakumara B.C., Shehzad S.A., Abbasi F.M. (2021). Bio-Marangoni convection flow of Casson nanoliquid through a porous medium in the presence of chemically reactive activation energy. Appl. Math. Mech..

[9] Kumar R.N., Gowda R.J.P., Madhukesh J.K., Prasannakumara B.C., Ramesh G.K. (2021). Impact of thermophoretic particle deposition on heat and mass transfer across the dynamics of Casson fluid flow over a moving thin needle. Phys. Scr..

[10] Jamshed W., Uma Devi S.S., Goodarzi M., Prakash M., Nisar K.S., Zakarya M., Abdel-Aty A.-H. (2021). Evaluating the unsteady Casson nanofluid over a stretching sheet with solar thermal radiation: An optimal case study. Case Stud. Therm. Eng..

[11] Kumar V., Madhukesh J.K., Jyothi A.M., Prasannakumara B.C., Khan M.I., Chu Y.-M. (2021). Analysis of single and multi-wall carbon nanotubes (SWCNT/MWCNT) in the flow of Maxwell nanofluid with the impact of magnetic dipole. Comput. Theor. Chem..

[12] Prasannakumara B.C. (2021). Assessment of the local thermal non-equilibrium condition for nanofluid flow through porous media: A comparative analysis. Indian J. Phys..

[13] Kumar R.N., Gowda R.J.P., Alam M.M., Ahmad I., Mahrous Y.M., Gorji M.R., Prasannakumara B.C. (2021). Inspection of convective heat transfer and KKL correlation for simulation of nanofluid flow over a curved stretching sheet. Int. Commun. Heat Mass Transf..

[14] Karvelas E.G., Karakasidis T.E., Sarris I.E. (2018). Computational analysis of paramagnetic spherical Fe3O4 nanoparticles under permanent magnetic fields. Comput. Mater. Sci..

[15] Ramesh G.K., Madhukesh J.K., Prasannakumara B.C., Roopa G.S. (2021). Significance of aluminium alloys particle flow through a parallel plates with activation energy and chemical reaction. J. Therm. Anal. Calorim..

[16] Chen S.-B., Shahmir N., Ramzan M., Sun Y.-L., Aly A.A., Malik M.Y. (2021). Thermophoretic particle deposition in the flow of dual stratified Casson fluid with magnetic dipole and generalized Fourier’s and Fick’s laws. Case Stud. Therm. Eng..

[17] Alhadhrami A., Alzahrani H.A.H., Kumar R.N., Gowda R.J.P., Sarada K., Prasanna B.M., Madhukesh J.K., Madhukeshwara N. (2021). Impact of thermophoretic particle deposition on Glauert wall jet slip flow of nanofluid. Case Stud. Therm. Eng..

[18] Hafeez A., Khan M., Ahmed J. (2021). Oldroyd-B fluid flow over a rotating disk subject to Soret–Dufour effects and thermophoresis particle deposition, Proceedings of the Institution of Mechanical Engineers. Part C J. Mech. Eng. Sci..

[19] Ashraf M., Abbas A., Ali A., Shah Z., Alrabaiah H., Bonyah E. (2020). Numerical simulation of the combined effects of thermophoretic motion and variable thermal conductivity on free convection heat transfer. AIP Adv..

[20] Epstein M., Hauser G.M., Henry R.E. (1985). Thermophoretic Deposition of Particles in Natural Convection Flow from a Vertical Plate. J. Heat Transf..

[21] Butt A.S., Tufail M.N., Ali A. (2016). Three-dimensional flow of a magnetohydrodynamic Casson fluid over an unsteady stretching sheet embedded into a porous medium. J. Appl. Mech. Technol. Phys..

[22] Raju C.S.K., Sandeep N., Babu M.J., Sugunamma V. (2016). Dual solutions for three-dimensional MHD flow of a nanofluid over a non-linearly permeable stretching sheet. Alex. Eng. J..

[23] Khan J.A., Mustafa M., Hayat T., Alsaedi A. (2014). On Three-Dimensional Flow and Heat Transfer over a Non-Linearly Stretching Sheet: Analytical and Numerical Solutions. PLoS ONE.

[24] Khan A., Khan D., Khan I., Ali F., Karim F.U., Imran M. (2018). MHD Flow of Sodium Alginate-Based Casson Type Nanofluid Passing Through A Porous Medium with Newtonian Heating. Sci. Rep..

[25] Devi S.P.A., Devi S.S.U. (2016). Numerical Investigation of Hydromagnetic Hybrid Cu—Al2O3/Water Nanofluid Flow over a Permeable Stretching Sheet with Suction. Int. J. Nonlinear Sci. Numer. Simul..

